# Gene Expression and miRNAs Profiling: Function and Regulation in Human Epidermal Growth Factor Receptor 2 (HER2)-Positive Breast Cancer

**DOI:** 10.3390/cancers11050646

**Published:** 2019-05-10

**Authors:** Rasha M. Sareyeldin, Ishita Gupta, Israa Al-Hashimi, Hamda A. Al-Thawadi, Halema F. Al Farsi, Semir Vranic, Ala-Eddin Al Moustafa

**Affiliations:** 1 College of Medicine, Qatar University, Doha 2713, Qatar; rasha.sareyeldin@gmail.com (R.M.S.); ishugupta28@gmail.com (I.G.); ia1507081@student.qu.edu.qa (I.A.-H.); halthawadi@qu.edu.qa (H.A.A.-T.); halfarsi@qu.edu.qa (H.F.A.F.); 2 Biomedical Research Centre, Qatar University, Doha 2713, Qatar

**Keywords:** breast cancer, HER2-positive breast cancer, biomarkers, microarray, gene expression profiling, miRNAs

## Abstract

Breast cancer is the second most common cause of cancer-related deaths among women worldwide. It is a heterogeneous disease with four major molecular subtypes. One of the subtypes, human epidermal growth factor receptor 2 (HER2)-enriched (HER2-positive) is characterized by the absence of estrogen and progesterone receptors and overexpression of HER2 receptor, and accounts for 15–20% of all breast cancers. Despite the anti-HER2 and cytotoxic chemotherapy, HER2 subtype is an aggressive disease with significant mortality. Recent advances in molecular biology techniques, including gene expression profiling, proteomics, and microRNA analysis, have been extensively used to explore the underlying mechanisms behind human breast carcinogenesis and metastasis including HER2-positive breast cancer, paving the way for developing new targeted therapies. This review focuses on recent advances on gene expression and miRNA status in HER2-positive breast cancer.

## 1. Introduction

Breast cancer is the most common type of cancer diagnosed in women worldwide with around 2.1 million new cases in 2018 according the World Health Organization. Breast cancer is the leading cause of mortality among women in the majority of countries worldwide [1], metastasis being the leading cause of death [2].

Although early detection of the disease through screening programs, education, and availability of therapeutic agents (chemotherapy, radiation, and targeted treatment) have led to an overall improvement, the survival rates are drastically reduced in 20–30% of patients who develop metastases. Metastatic breast cancer still represents an incurable disease with a poor outcome, as the median survival is 2–4 years depending on the breast cancer subtype [3].

Several risk factors can be identified at the onset of breast cancer including age at menarche [4], null parity [5], postmenopausal obesity [6], late menopause, high hormonal levels including estradiol, prolactin, and insulin-like growth factor [7,8].

A recent study has shown that breast cancer heterogeneity extends beyond the histopathological classification; however, patients with breast cancer are still grouped according to clinicopathological criteria in order to decide the appropriate therapy and predict their prognosis. These criteria include patient age, tumor size, histological grade as well as the presence or absence of lymph nodes and distant metastases, in addition to hormone receptor, estrogen (ER), progesterone (PR) and human epidermal growth factor receptor 2 (HER2) status [9]. Although this has successfully led to decreasing mortality rates over the last three decades, it failed to predict the outcome in different patients, as patients with the same tumor features may have completely different outcomes [9] while other patients suffer from significant toxic side effects [10]. Thus, accurate stratification of breast cancers into clinically related subtypes is of major importance for therapeutic decision making [11].

The availability of high-throughput technology for gene expression profiling, such as microarray, quantitative, and differential-display reverse transcription polymerase chain reaction (RT-PCR), as well as next generation sequencing, have shown that tumor cells respond differently to treatment which is not resolved by clinicopathological aspects, rather by intrinsic molecular features that can be explored using molecular technology [12]. Gene expression (mRNA) profiling studies have allowed intrinsic classification of breast cancer into five main subtypes: Luminal A (ER+/PR+/HER2−) are usually of low grade, luminal B (ER+/PR−/+/HER2+/−) are generally of higher grade with higher proliferation rate, normal-like subtype that resembles normal breast tissue and is associated with good prognosis, triple-negative breast cancer (ER−/PR−/HER2−), and HER2-enriched subtype (ER−/PR−/HER2+) [13,14,15]. On the other hand, miRNA’s signatures can further subclassify breast cancer [16], leading to the identification of new molecular subtypes [17].

In this review, we will focus on novel prognostic and predictive markers of HER2-positive breast cancer subtype. HER2-positive breast cancer represents 15–20% of breast cancer cases [18,19] and is defined by the overexpression of HER2 protein as characterized by immunohistochemistry (IHC) status or by florescence or chromogenic in situ hybridization (FISH/CISH) of *HER2* gene copy number or a *HER2/CEP17* ratio of 2 or greater [20]. This type of cancer is associated with poor prognosis, short survival, and high rates of recurrence [20].

## 2. Human Epidermal Growth Factor Receptor (EGFR) Family

EGFR are transmembrane receptors formed of three parts: An extracellular ligand binding site, a transmembrane part, and an intracellular tyrosine kinase domain that comprises of HER1, HER2, HER3, and HER4 [21]. The human epidermal growth factor receptor 2 (HER2) is a 1255 amino acid, 185 kD localized on the long arm of chromosome 17q [22,23]. Activation of HER1, HER3, and HER4, occur through several ligands [24], including transforming growth factor alpha (TGF-α), amphiregulin (for EGFR), EGF, and neuregulins (for HER3 and HER4) [25]. These signaling proteins control several cellular functions including cell proliferation, migration, differentiation, angiogenesis, and survival [26] via the downregulation of second messenger pathways as well as through cross talk with other membrane signaling pathways [25,27,28].

HER2 receptor is highly expressed in human tissues including the cell membranes of epithelial cells in the gastrointestinal, respiratory, reproductive, and urinary tract as well as in the skin, breast, and placenta [29]. Amplification or overexpression of *HER2* oncoprotein plays an important role in the pathogenesis of various solid tumors [30], including upper gastrointestinal tract (stomach and gastroesophageal junction adenocarcinoma) [31], ovarian cancer, colon, salivary gland [32], lung cancer, and breast cancer [33].

## 3. HER2-Positive Breast Cancer

Also known as HER2-enriched breast cancer, HER2-positive breast cancer represents 15–20% of all breast cancers [18,19] and are dependent on the high expression of HER2 oncoprotein, and its intensive downstream signaling pathways [21].

**Figure 1:**
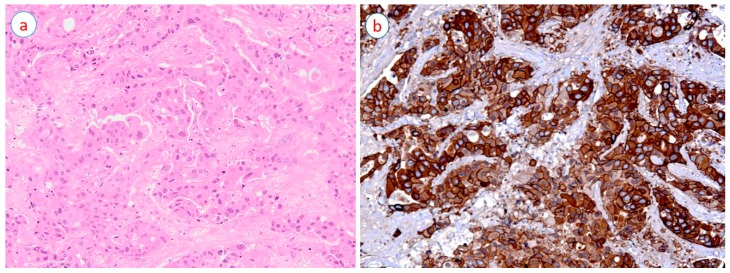
A case of high-grade invasive breast carcinoma (hematoxylin and eosin stain) (**a**) with diffuse (100% of cancer cells) and strong (3+ intensity) human epidermal growth factor receptor 2 (HER2) expression (10×) (**b**).

The primary mechanism of HER2 activation in breast cancer is its gene amplification on the long arm of chromosome 17 (17q12-21-21.32) [34]; this consequently leads to the overexpression of *HER2* protein (receptor) (Figure 1b) causing homo- or heterodimerization with other HER family members and resulting in auto- and transphosphorylation, which in turn activates several signaling pathways [19]. Among the most important pathways activated by *HER2* overexpression is the phosphatidylinositol 3-kinase (PI3K), mammalian target of rapamycin (mTOR) axis, which are responsible for the regulation of important cellular functions including cellular metabolism, migration, as well as proliferation and angiogenesis [35,36,37]. Another important pathway activated by *HER2* overexpression is the Ras/Raf/MEK/ERK pathway (also known as extracellular signal-regulated kinase/mitogen-activated protein kinase (ERK/MAPK) pathway) [19].

It is worth noting that HER2 status of breast cancer can change during the disease progression from negative to positive, one plausible reason being the oncogenic *HER2* amplification during cancer progression [38,39,40,41].

Recent studies have also described *HER2* mutations in a subset of breast cancers as well as other malignancies [42,43]. These mutations appear to be activating and driving breast carcinogenesis [44]. A systematic review of Petrelli et al. [42] revealed that the frequency of *HER2* mutations in breast cancer is ~3% with most of these affecting the intracellular (kinase) domain of HER2 receptor. Notably, *HER2* mutations predominantly affect HER2-negative breast cancers (only 30% are HER2 positive (amplified)) while 63% of breast cancers are ER+ [42]. *HER2* mutations in breast cancer are also associated with poor outcomes [45].

HER2-positive breast cancers are morphologically poorly differentiated with a marked pleomorphism and a high proliferation rate (high grade cancers) (Figure 1a); they are prone to lymph node metastasis, tend to show a degree of resistance to certain chemotherapeutic agents [46], and have a higher rate of recurrence and distant metastasis, causing a high mortality rate [47].

Although approximately 50% of all HER2-positive breast cancers express the steroid receptors estrogen (ER) or progesterone (PR) (luminal B breast cancers), they are usually resistant to endocrine treatment, particularly tamoxifen [48]. There is a growing clinical evidence that suggests the presence of a molecular crosstalk between ER and HER2 pathways [49,50], indicating ER+/HER2+ breast cancers as a distinct breast cancer subtype that may require a specific approach in treatment [51].

## 4. Treatment of HER2-Positive Breast Cancer

HER2-positive breast cancer is targeted by personalized therapy using monoclonal antibodies, such as trastuzumab (Herceptin), this treatment targets the HER2 receptor and blocks the related pathways inhibiting proliferation and survival as well as migration and cell invasion, leading to prolonged patient survival [52]. Moreover, other treatments such as lapatinib [53], pertuzumab as well as ado-trastuzumab emtansine (T-DM1) have also been approved for use in the treatment of HER2-positive breast cancer, particularly in metastatic setting [54]. Despite the availability of targeted therapy, almost 40% of patients with metastases develop primary resistance to trastuzumab and others, as approximately 60% of patients present with acquired resistance following one year of treatment [55].

Early diagnosis of breast cancer, as well as monitoring disease progression and its response to treatment, is critical in the management of this disease [56]. However, existing biomarkers and diagnostic tools, such as carcinoembryonic antigen (CEA) and carbohydrate antigens (CA), are of low sensitivity [57], therefore, clinicians cannot rely on these measures as screening tools. An ideal biomarker should be obtained noninvasively and be highly sensitive to detect the tumor as early as possible [58]. This new approach can have a major impact on the clinical management including breast cancer classification, prognosis, predicting therapy outcome, as well as follow-up after surgery and prediction of metastasis, and tumor recurrence.

## 5. Gene Expression Profiling of HER2-Positive Breast Cancer

Progress in microarray technology led to gene expression profiling of breast cancer with the aim of identifying patients that can benefit from adjuvant chemotherapy, and as a prognosis predictor in cancer patients [59,60]. HER2-positive breast cancers are a heterogeneous subgroup in which the resistance to treatment has been associated with specific gene expressions or gene mutations [61]. A recent study reported a genomic characterization of 64 HER2-positive breast cancer genomes. Based on genomic features including somatic mutations, copy-number changes, or structural variations, HER2-positive breast cancers are characterized into four subgroups (Groups A, B, C, and D). While subgroups A and B are ER+ and close to luminal B intrinsic subtype with low tumor protein p53 (TP53) values and amplification of cyclin D1 (*CCND1*) and ribosomal protein S6 Kinase B1 (*RPS6KB1*), groups C and D are ER− and close to the HER2-positive intrinsic subtype with high TP53 expression [61]. Moreover, a set of 20 key genes are classified according to their expression levels. Of these 20, 13 genes (Dickkopf-1 (*DKK1*), matrix metalloproteinase 15 (*MMP15*), baculoviral IAP repeat-containing 5 (*BIRC5*), *CCND1*, origin recognition complex subunit 6 homolog-like (*ORC6L*), *MKi67*, cyclin E1 (*CCNE1*), *TP53*, *HER1/EGFR*, ATPase H+ transporting V0 subunit a4 (*ATP6V0A4*), prolyl endopeptidase (*PREP*), reticulon 4 interacting protein 1 (*RTN4IP1*), kinesin-like protein (*KIF18A*)) are upregulated in HER2-positive breast cancer cells and correlated with poor survival rates because of their role in promoting proliferation, progression, lymph node, and metastasis (bone, lung, liver, and brain), and contributing to tumor aggressive behavior in colony growth. On the contrary, genes including phosphatidylinositol-4,5-bisphosphate 3-kinase catalytic subunit alpha (*PIK3CA*), phosphatase and tensin homolog (*PTEN*), inositol polyphosphate 4-phosphatase type II (*INPP4B*), phosphatidylinositol 3-kinase regulatory subunit (*PIK3R1*), *TP63* are downregulated and resulted in poor prognosis and survival. In addition, several genes are studied as targets of recently developed drugs (Table 1). Overexpression of genes including post-GPI attachment to proteins 3 (*PGAP3*) [11] and growth factor receptor-bound protein 7 (*GRB7*) [11,14] are also associated with *HER2* amplified breast cancers. While, 40–80% HER2-positive breast cancer cases are known to harbor *TP53* mutations [62,63].

HER2-positive breast cancer patients generally benefit from neoadjuvant chemotherapy in the form of anthracyclines and taxanes with a higher complete pathological response in comparison with luminal and triple-negative subtypes [80]. Although molecular targeting agents such as trastuzumab are available, not all patients respond to it. The underlying mechanisms for resistance to trastuzumab still lies nascent, but few studies showed activation of the PI3K pathway [81] to be involved in drug resistance [82]. Activation of PI3K pathway through *PIK3CA* mutations, loss of *PTEN* [83] or C-X-C motif chemokine receptor type 4 (*CXCR4*) upregulation [80] can lead to cell growth through mTOR-mediated signaling [82]. Another study explored the genomic features of patients involved in a phase I/II study [84] using GeneChip microarray [82]. They found that mutations in B-Raf proto-oncogene, serine/threonine kinase (*BRAF*); *EGFR1*; *PIK3CA*; and proto-oncogene, receptor tyrosine kinase (*KIT*) are more frequent in trastuzumab-resistant HER2-positive metastatic breast cancer. These mutations may upregulate MAPK/ERK pathway and consequently induce resistance to trastuzumab. Furthermore, use of OncoScan^tm^ detected mutations in five genes (catenin beta 1 (*CTNNB1*); HRas proto-oncogene, GTPase (*HRAS*); KRAS proto-oncogene, GTPase (*KRAS*); neurofibromin 2 (*NF2*); and SWI/SNF related, matrix associated, actin dependent regulator of chromatin, subfamily b, member 1 (*SMARCB1*) in HER2-positive breast cancer [82]. This study showed that trastuzumab-resistant ER+/HER2+ breast cancers carry genetic alterations that affect the DNA repair mechanism while trastuzumab-resistant ER−/HER2+ breast cancers carry mutations that activate mitotic signaling [82]. Another study analyzed hormone receptor (HR) status in HER2-positive breast cancer [85]. They used gene expression analysis to distinguish between HR−/HER2+ and HR+/HER2+. The authors found 127 differentially expressed genes [85]. The results of this investigation revealed that 83% of these genes are upregulated in HR−/HER2+ cohort of which 41 genes belong to the PI3K pathway, 26 genes are involved in transcriptional regulation and 22 genes are associated with MAPK pathway. This clearly indicates an active role of PI3K, and MAPK signaling pathways in HR−/HER2+ group [85].

## 6. MicroRNA

MicroRNAs (miRNAs) are a class of small regulatory, noncoding RNA molecules measuring approximately 18–25 nucleotides in length [86]. Since their initial discovery in *Caenorhabditis elegans*, they have been shown to play a major role in gene expression modulation and controlling major pathways [86].

miRNAs regulate the expression of 30–60% of human genes and are important modulators of cell proliferation, cell differentiation, cell development, cell-cycle progression, angiogenesis, epithelial-mesenchymal transition, stem cell renewal, apoptosis as well as cell migration, invasion, and metastasis [87]. Dysregulation in miRNA expression is associated with multiple human diseases such as cancer [88,89] and can act as promotor (oncomiR) or suppressors of tumorigenesis (antioncomiR) [90].

Recent studies showed that miRNAs can be detected not only in tissues but also in body fluids such as blood, serum, and urine, thus indicating miRNAs to be easily accessible without the need for invasive procedures [91]. Since miRNAs are highly stable, easily detectable in sera and can be measured easily by different techniques (deep sequencing, microarray as well as RT-qPCR) [87], they appear to be ideal biomarkers for diagnosis, follow-up, and prognosis prediction of cancer patients [92].

In the last decade, healthcare systems have greatly evolved, they have integrated point of care (POC) diagnostics as an integral part of their transformation [93]. POC allows the clinician to perform laboratory tests near the patient with quicker results, instead of conducting the tests in a routine laboratory set away from the patient, thus contributing to better patient care [94]. Since, the conventional assays (Northern blot, microarray assay, in situ hybridization, RT-qPCR, and next generation gene sequencing) are far from achieving the POC in breast cancer, development of miRNAs POC diagnostic tools and assays is a promising field of research in the upcoming decade as it can maximize diagnostic, prognostic, and predictive benefits for the patient [95,96,97,98]. New amplification and miRNAs detection assays are further being developed to fit criteria for POC diagnostics, the most promising ones being based on nanotechnology; however, they are yet to be integrated in clinical setting [93].

Numerous miRNAs have been reported to be dysregulated in human malignancies including lung, breast, ovarian, bladder, colon, and other types of carcinoma. Therefore, it is reasonable to assume that dysregulation in the miRNA machinery is a plausible reason for the onset and progression of human cancers [99]. Accumulating evidence indicates that miRNAs are dysregulated in all stages of breast cancer and thus can be used as diagnostic as well as prognostic and predictive biomarkers [100,101].

Previous studies reported that different breast cancer subtypes display various molecular miRNA signatures [16,100,102]. miRNAs have been dysregulated in breast cancer as shown by profiling breast tissues from healthy individuals and those from breast cancer patients [87]. Several miRNAs (miR-21, miR-10b, miR-155, and Let-7a) are found to be dysregulated in sera of breast cancer patients compared to healthy individuals [92,103,104,105]. Different miRNAs have been associated with specific breast cancer subtypes, specifically, Let-7f, Let-7c, and miR-10a are associated with luminal A, and miR155, miR-93, miR-18a, and miR-135b are associated with basal subtype [100]. On the other hand, miR-150 and miR-142-3p are associated with HER2-positive subtype [100], while miR-153, miR-10b, miR-26a, and miR146a, are shown to be potential biomarkers of triple negative subtype [106].

## 7. MicroRNA and HER2-Positive Breast Cancer

To date, only few studies have described the regulation of HER family receptors by miRNAs. Therefore, diagnostic and prognostic roles of miRNAs are still nascent and require further investigations [107].

A few studies have shown that miRNAs have a diagnostic role in HER2-positive breast cancer. miRNAs have been involved in the expression of 26 proteins within the EGFR1 signaling pathway. Among these, miR-147, miR-124, and miR-193-3p have been shown to act as tumor suppressors for the EGFR1 pathway, inhibiting the proliferation of HER2-positive breast cancers [108]. Another study identified 38 miRNAs inhibiting HER2 signaling and cell growth; the most vital miRNAs included miR-342-5p, miR-634, miR-491-5p, and miR-637 [109]. Furthermore, other miRNAs including miR-331-3p, miR-541, miR-134, miR-453, miR-193a-5p, miR-498, and miR-552 have also been identified as regulators of 3′UTR of *HER2* gene [109]. In comparison with HER2-negative breast cancers, the expression of two miRNAs (miR-342-5p and miR-744) was lost in HER2-positive breast cancer [109]. Another study identified downregulated expression of 43 miRNAs in HER2-positive breast cancer compared with HER2-negative breast cancer, indicating loss of miRNAs in HER2-positive breast cancers. This study further identified seven miRNAs specific to HER2-positive breast cancer. These include Let-7f, Let-7g, miR-107, miR-10b, miR-126, miR-154, and miR-195 [110]. A signature of 5 miRNAs (miR-520d, miR-181c, miR-302c, miR-376b, and miR-30e-3p) predicting HER2 status in patients with early breast cancer was also identified [111]. This study also revealed that miR-30b is differentially expressed between HER2-positive and HER2-negative breast cancers [111].

Furthermore, miRNAs also have a prognostic role in HER2-positive breast cancer. miRNA machinery elements, including Dicer, Drosha, DGCR8, Argonaut, and TRBP, and are reported to be involved in the progress of several cancer subtypes [99]. Thus, a study by Grelier et al. [112], suggested that *DICER1* is significantly lower in the HER2-positive, luminal B, and basal-like subtypes and can be used as a predictive marker for metastases-free survival [16,112]. Another study conducted by Dedes et al. (2011) [113], reported the loss of *DORSH* to be associated with *HER2* gene amplification and protein overexpression [113].

MicroRNA profiling has helped in enhancing breast cancer classification and in stratifying patients according to their response to therapy [114]. Du et al. [115] utilized miRNA microarrays and identified nine differentially expressed miRNAs between recurrent and nonrecurrent breast cancer patients. They developed a 2-miRNA (miR-4734 and miR-150-5p)-based prognostic signature that can be a reliable prognostic biomarker for patients with HER2-positive breast cancer [115]. This signature successfully classified patients into two groups based on the risk of tumor recurrence, independent of clinical characteristics, and predicted the five-year disease-free survival comparatively better than other clinicopathological factors, thus adding prognostic value to the TNM staging system [115]. While elevated miR-150 expression is associated with poorer clinical outcome in nonsmall-cell lung cancer [116] as well as triple negative breast cancer [117], miR-4734 has been recently identified in breast cancer by extensive next-generation sequencing analysis and encodes within the *ERBB2/Her2* gene, which is upregulated in HER2-positive breast cancer patients [118].

Recent studies using relative real-time PCR analyzed differential expression of potential miRNAs to U6 RNA (noncoding small nuclear RNA which is conserved in all species [119]) in trastuzumab-resistant and trastuzumab-sensitive breast cancer cells [120]. The study showed that miR-23b-3p, miR-195-5p, miR-656-5p, and miR-340-5p are significantly dysregulated in trastuzumab-resistant cells and their potential targets are involved in drug resistance pathways (MAPK, PI3K-AKT, and FOXO). [120]. These miRNAs may be involved in the underlying mechanism for trastuzumab resistance, thus targeting these regulatory networks may help overcome trastuzumab resistance; however, further research is required to elucidate the clinical relevance of these observations [120].

One of the best characterized miRNAs in HER2-positive breast cancer is miR-21 [121]. It is located on chromosome 17-q 23.2 and may serve as a diagnostic biomarker for early breast cancer [122]. miR-21 is located on the intron of the protein coding gene *TMEM49*, however it was reported that miR-21 mostly has its own transcriptional system and is regulated independently [123]. The miR-21 promotor region contains several enhancer elements including sites that allow it to bind to activation protein 1 (AP-1), Nuclear factor-1 (NF1), SRF, TP53 and STAT3 (signal transducer and activator of transcription 3) [123]. Fujito et al. demonstrated, using the ChIP assays, that AP-1 stimulated the transcription of miR-21 initiating downregulation of NF1B1 expression. In addition, NF1B bound to the promotor region of miR-21 and negatively regulated it [123], implying that through this double-negative feedback loop miR-21 expression is sustained [124].

**Figure 2:**
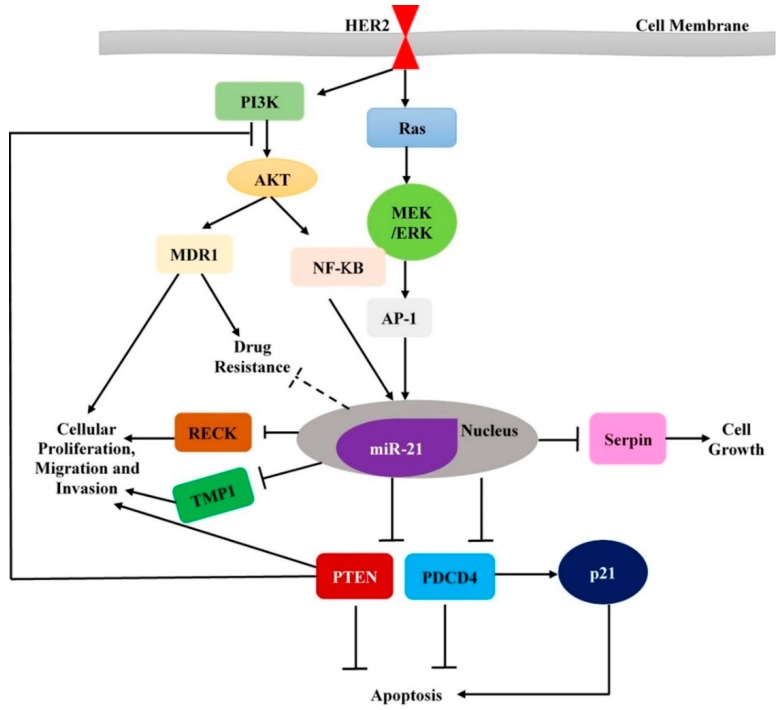
A schematic representation of the regulatory network displaying HER2-regulators-miRNAs-targets (and crosstalk with canonical HER2 targets)-phenotypes.

Previous studies have demonstrated that miR-21 acts as an oncogene by targeting tumor suppressor genes *PDCD4* (programmed cell death 4), *TPM-1* (tropomycin 1), and *PTEN* (phosphatase and tensin homolog) [125,126,127] (Figure 2). miR-21 is overexpressed in breast tumor tissues as well as in sera of patients when compared with controls [105]. In addition, *PDCD4* expression is lost by the presence of miR-21 [128] in HER2-positive breast cancer cells [125]. *PDCD4*, a target of miR-21, is a regulator of AP1 and also induces the expression of cyclin dependent inhibitor (cdk) 21 [129].

miR-21 also enhances epithelial-mesenchymal transition, thus promoting progression of primary HER2-positive breast cancer [130]. Loss of PTEN stimulates IL-6, leading to upregulation of miR-21 thereby inducing EMT, which could be activated by PI3K and STAT3/NF-κB pathways [130] (Figure 2). Another study reported the role of *TGF-β* in upregulating miR-21 expression in vascular smooth muscles (VSMCs), as well as in MDA-MB-468 breast cancer cells, through the SMAD signal pathway [131]. Another recent study conducted by Xiaomeng Dai et al. [132], demonstrated that upregulation of miR-21 in response to *TGF-β*, is associated with chemoresistance and cell invasion in vitro. In addition, inhibition of *PTEN* expression was found to be mediated by *TGF-β* inducing increased expression of miR-21 in breast cancer cells; however, treatment of these cells with miR-21 inhibitor, restored *PTEN* expression [132]. miR-21 was also implicated in cell invasion and metastases in HER2-positive breast cancer. Overexpressed HER2 enhances the expression of miR-21, which could promote cell invasion of HER2-positive breast cancer through the stimulation of MAPK pathway [125].

miR-21 expression is enhanced via MAPK (ERK1/2) pathway upon activation of HER2/neu signaling in breast cancer cells [125]. A study conducted by Hatley et al., provided further evidence that miR-21 targeted the RAS pathway and activated the RAS/MEK/ERK pathway [133]. Therefore, miR-21 is considered a critical downstream player involved in HER2/neu-RAS-MEK-ERK signaling pathways that is majorly linked with the onset of various tumors including breast cancer [125]. Stimulation of *ID-1* expression, enhanced by miR-21 upregulation, promote cell invasion as well as enhance oncogene signaling pathways associated with MEK-ERK pathway activation [134]. Moreover, since members of EGFR family, including HER1, HER2, HER3, or HER4, are known to stimulate the MEK-ERK pathway [135,136], it can plausibly be involved in the enhanced expression of miR-21 in breast cancer cells.

MiR-21 was also found to significantly affect the response to neoadjuvant therapy in HER2-positive breast cancers [130]. DNA-damage due to chemotherapeutic drugs elevated miR-21 expression by stimulating NF-κB, allowing breast cancer cells to escape DNA damage-induced apoptosis thus promoting their invasiveness [137]. This indicates overexpression of miR-21 can lead to resistance to trastuzumab-chemotherapy treatment in patients with HER2-positive breast cancer [130]. Another in vitro (SKBR3, BT474, and MDA-435) and in vivo (mammary fat pad of mice) study, showed that overexpression of miR-21 is correlated with poor response to trastuzumab in HER2-positive breast cancers [138]. In addition, it was shown that miR-21 is not associated with drug response in HER2-negative patients. Taken together, the body of evidence indicates the important role of miR-21 as a predictive biomarker for resistance to treatment (trastuzumab and cytotoxic drugs, such as cisplatin and paclitaxel) in HER2-positive patients [130].

Another important miRNA, miR-210, is located on chromosome 11p15.5 [139]. miR-210 levels are elevated in the plasma of HER2-positive breast cancer patients, and are associated with trastuzumab resistance as well as with tumor presence, lymph node metastases, and poor survival. It is also involved in tumor progression by targeting FBXO31 [140,141,142]. Therefore, it has been suggested that plasma levels of miR-210 can be useful in predicting and monitoring response to trastuzumab [143].

miR-489, located on chromosome 7q21.3 [144] has been described as one of the potential targets that downregulates HER2 signaling pathway, although the underlying mechanism remains nascent [109]. miR-489 expression is significantly decreased in HER2-positive breast cancers compared with luminal subtypes. Loss of miR-489 expression is observed in HER2-positive patients in comparison with normal breast tissues from the same patient [144]. Recent investigations indicated that elevated expression of miR-489 blocked cell growth, invasion and EMT by targeting *Shp2* in hypopharyngeal carcinoma [145], *SMAD3* in breast cancer [146], *AKT3* in ovarian cancer [147], and *Dek* in ovarian cancer, as well as muscle stem cells [147,148]. Furthermore, *Shp2* was seen to enhance the RAS-MAPK pathway regulating breast cancer proliferation [144]. miR-489 is a candidate miRNA that can be associated directly with the 3′-UTR of *HER2* mRNA downregulating its expression [144]. miR-488 expression level is correlated with more aggressive tumor phenotype among HER2-positive breast cancers [144]. Furthermore, downregulated miR-489 enhances cancer cells resistance to chemotherapeutic drugs [149]; indicating that loss of miR-489 plays a role in tumor development and in anticancer drug sensitivity by regulating different target genes. Dysregulated expression of miR-489 enhances *Smad3* expression activating EMT-like properties, thus conferring chemo-resistance [146]. Targeting miR489 as well as *Smad3* [146] can be of substantial use in treatment of HER2-positive breast cancer [144]. Unregulated expression of miR-489 can aid to control aggressiveness of HER2-positive breast cancer.

Table 2 below summarizes key miRNAs with their expression levels and biological functions in HER2-positive breast cancer. Table 3 provides more details on the cellular position of miRNAs (intracellular or extracellular).

## 8. Clinical Relevance of miRNAs in HER2-Positive Breast Cancer

As mentioned above, miRNAs have been involved in the onset and progression of breast cancer and have the ability to reverse resistance to drugs like tamoxifen; as studies have shown that re-expression of miR-375 [169], miR-342 [170], and miR-449a [171] suppressed tamoxifen resistance; indicating them to be potential biomarkers for therapeutic strategies. miR-210 levels in plasma are linked with trastuzumab resistance in HER2-positive breast cancer patients [172]. A recent study showed that enhanced levels of miR-770-5p interfere with trastuzumab effectiveness; increased miR-770-5p levels downregulated the total or phosphorylated levels of AKT and ERK, that are the two main regulator proteins of PI3K and MAPK signaling pathways [173]. The study further indicated a synergistic role of miR-770-5p with tyrosine kinase inhibitors as an effective therapeutic strategy for breast cancer; however, further research is needed to confirm the efficacy of the combination in vivo [173]. Another study identified a tumor suppressive role of miR-1296-5p in HER2-positive breast cancer cells; increased miR-1296-5p levels reduced its target protein level and mTORC1/S6 activation; thus, miR-1296-5p is able to block cellular proliferation of HER2-positive breast cancer cells and increase their sensitivity to cisplatin and 5-fluorouracil-induced apoptosis [174]. A very recent study by Yang et al., [175] identified three upregulated miRNAs (miR-200b, miR-135b, and miR-29a) and one downregulated miRNA (miR-224) in trastuzumab-resistant breast cancer samples in comparison to trastuzumab-sensitive breast cancers. The study provided further evidence that miRNAs may be reliable biomarkers of the response to anti-HER2 drugs [175]. Another study identified two downregulated miRNAs (miR-141 and miR-375) in trastuzumab-resistant breast cancer cells [176]. Furthermore, silencing of miR-141 enhanced ERBB4 expression, which plays a critical role in trastuzumab resistance in breast cancer cells; taken together, these results indicate that both miR-141 as well as its target, ERBB4, may be potential tools for the efficient treatment of trastuzumab-resistant breast cancers [176].

On the other hand, amplification in the 17q23 region (amplicon) leads to dysregulated expression of oncogene *WIP1* and oncomiR, miR-21; both of which have been involved in promoting breast tumorigenesis as well as resistance to anti-HER2 therapies [177]. Research by Lui et al., (2018) [177] showed that knockdown of both *WIP1* and miR-21, reduced proliferation, survival, and tumorigenic potential of HER2-positive breast cancer cells harboring 17q23 amplification, thus, providing an effective therapeutic strategy for HER2-positive breast cancers [177]. Development of pH-sensitive nanoparticles that are capable of capturing *WIP1* and miR-21 inhibitors will aid in paving the way for effective future therapeutic approaches against trastuzumab-resistant breast cancers [177].

miRNAs are usually present in microvesicles or bound to lipoproteins in the blood [178]; while several miRNAs in body fluids are concentrated in exosomes [179]. Recently, exosomal miRNAs in body fluids have been shown to play a diagnostic role in several cancers including breast [180,181,182]. Cancer patients frequently display increased levels of tumor-derived exosomes in plasma or serum compared with those in healthy donors [183]. A recent study identified 11 exosomal miRNAs (miR-338-3p, miR-340-5p and miR-124-3p, miR-29b-3p, miR-20b-5p, miR-17-5p, miR-130a-3p, miR-18a-5p, miR-195-5p, miR-486-5p, and miR-93-5p) in serum of patients associated with breast cancer recurrence [183]. Another study revealed differential expression of exosomal miR-101 and miR-373 between patients with breast cancer and benign breast tumors; exosomal miR-101 serum levels were dysregulated in HER2-positive breast cancer compared with their levels in healthy women [184]. Studies have also shown that lack of estrogen stimulation may significantly increase miR-101-mediated activation of the Akt signaling pathway [185], thus promoting increased cell survival and malignant progression of breast cancer [186]. Research focusing on the underlying mechanism of secretion of miRNAs into exosomes or the retention of miRNAs inside cancer cells is still poorly understood. Further clinical and functional validation studies are essential for future application of these miRNAs.

## 9. Conclusions

HER2-positive breast cancer is an aggressive subtype of breast cancer with a poor outcome despite available targeted treatment modalities. There is an increasing body of evidence that various gene and miRNA signatures are specific for HER2-positive breast cancer. None of these has become fully clinically applicable and therefore, further studies are necessary for their validation. More specifically, there is a huge gap regarding the miRNA profile of HER2-positive breast cancer, therefore, it is necessary to conduct more in-depth studies, which may lead to specific miRNA markers or targets for HER2-positive breast cancers.

On the other hand, it is important to develop an animal model for HER2-positive breast cancer, which can be used to study the miRNA and gene profile of the main metastatic sites of HER2-positive breast cancer metastasis, more specifically, brain and lung. Such a study may allow to develop specific markers for metastatic HER2-positive breast cancers.

## Figures and Tables

**Table 1:** Gene expression profiling in HER2-positive breast cancer.

Gene	Role in HER2-Positive Breast Cancer Progression	References
*DKK1*	High levels correlate with poor prognosis, contribute to lymph nodes metastasis and bone metastasis	[64,65,66]
*MMP15*	Highly expressed in HER2-positive breast cancer cells and mediate tumor progression	[64,67]
*BIRC5*, *CCND1*, *ORC6L*, *MKi67*, *CCNE1*	Highly expressed in HER2-positive cancer cells and promote proliferative activity of cancer and contribute to tumor aggressive	[64,68,69]
*TP53*	Highly expressed (mutated) in HER2-positive tumor cells and contribute to early onset and progression of HER2-positive breast cancer cells.	[70,71]
*BCL2*	Upregulated in HER2-positive breast cancer, considered as a diagnostic marker since it inhibits apoptosis and promotes colony growth.	[70,72]
*HER1/EGFR1*	Overexpressed and contribute to increased tumor size and poor progression-free prognosis.	[70,73]
*PIK3CA*, *PTEN*, *INPP4B*	Downregulated (mutated) in HER2-positive breast cancer cells, contribute to tumor growth, cell proliferation, and poor survival outcomes	[70,74]
*PIK3R1*	Underexpressed (mutated) in HER2-positive breast cancer cells promoting metastasis and contributing to poor metastasis free survival	[70,75]
*ERBB2*	It is the target of trastuzumab in HER2-positive breast cancer cells. It is amplified in 15–20% of all breast cancers; activating mutations are present in ~3% of breast cancers	[76,77]
*ATP6V0A4*, *PREP*, *RTN4IP1*, *KIF18A*	Upregulated in HER2-positive breast cancer cells, contribute to visceral metastasis and poor overall survival	[78]
*TP63*	Downregulated in HER2-positive rich breast cancer cells, inhibits brain metastasis	[79]

**Table 2:** A list of miRNAs and their roles in HER2-positive breast cancer.

Biological Functions	miRNAs		References
	Stimulate	Inhibit	
Cell proliferation	miR-96, miR-96-5p, miR-10b, miR-143, miR-127-3p, miR-19a, miR-222-3p	miR-335-5p, miR-376a-3p, miR-452, miR-182, miR-377-3p	[109,141,142,147,150,151,152,153,154,155]
Tumor metastases and progression	miR-96, miR-96-5pm miR-10b, miR-127-3p, miR-320, miR-19a, miR-221, miR-221-3p, miR-17, miR-222-3p, miR-9-5p	miR-148a, miR-148a-3p, miR-148b-3p, miR-335-5p, miR-376a-3p, miR-452, miR-182, miR-377-3p	[109,141,142,147,150,151,153,154,155,156,157,158,159]
Cell Apoptosis	miR-148a, miR-148a-3p, miR-148b-3p, miR-376a-3p, miR-452, miR-468	miR-221, miR-221-3p	[109,141,142,153,156,157]
Resistance to therapy	miR-200, miR-200c, miR-221, miR-100, miR-222-3p, miR-9-5p		[142,156,159,160]

**Table 3:** Localization of miRNAs.

miRNAs	Intracellular/Extracellular	References
miR-96	Extracellular	[161,162]
miR-96-5p	Extracellular and Intracellular	[87,163]
miR10-b	Extracellular and Intracellular	[164]
miR-143	Extracellular and Intracellular	[164]
miR-127-3p	Extracellular	[87,165]
miR-19-a	Extracellular and Intracellular	[164]
miR-7	Extracellular	[161,165]
miR-148-a	Extracellular	[164]
miR-200c	Intracellular	[166]
miR-100	Intracellular	[164]
miR-452	Extracellular and Intracellular	[164]
miR-182	Extracellular and Intracellular	[87,167]
miR-148a	Extracellular	[164]
miR-148b-3p	Extracellular	[87,168]
miR-221	Intracellular	[110,161]
